# Advances in the Progression and Prognosis Biomarkers of Chronic Kidney Disease

**DOI:** 10.3389/fphar.2021.785375

**Published:** 2021-12-21

**Authors:** Zhonghong Yan, Guanran Wang, Xingyang Shi

**Affiliations:** ^1^ Heilongjiang University of Chinese Medicine, Harbin, China; ^2^ Department of Nephrology, First Teaching Hospital of Tianjin University of Traditional Chinese Medicine, Tianjin, China

**Keywords:** biomarkers for early diagnosis, progression biomarkers, prognostic biomarkers, chronic kidney disease, advances

## Abstract

Chronic kidney disease (CKD) is one of the increasingly serious public health concerns worldwide; the global burden of CKD is increasingly due to high morbidity and mortality. At present, there are three key problems in the clinical treatment and management of CKD. First, the current diagnostic indicators, such as proteinuria and serum creatinine, are greatly interfered by the physiological conditions of patients, and the changes in the indicator level are not synchronized with renal damage. Second, the established diagnosis of suspected CKD still depends on biopsy, which is not suitable for contraindication patients, is also traumatic, and is not sensitive to early progression. Finally, the prognosis of CKD is affected by many factors; hence, it is ineviatble to develop effective biomarkers to predict CKD prognosis and improve the prognosis through early intervention. Accurate progression monitoring and prognosis improvement of CKD are extremely significant for improving the clinical treatment and management of CKD and reducing the social burden. Therefore, biomarkers reported in recent years, which could play important roles in accurate progression monitoring and prognosis improvement of CKD, were concluded and highlighted in this review article that aims to provide a reference for both the construction of CKD precision therapy system and the pharmaceutical research and development.

## Introduction

CKD is one of the non-communicable diseases that, in a condition of persistently reduced kidney function over 3 months, is considered a serious threat to folk health with high morbidity on a global scale (Carney, 2020). With different pathogenesis, CKD gradually progresses to irreversible nephron loss, which results in end-stage renal disease (ESRD) (Ruiz-Ortega et al., 2020). A recent Global Burden of Diseases, Injuries, and Risk Factors Study, reported in Lancet in 2020, indicated that 1.2 million people died from CKD in 2017, the 12th leading cause of death worldwide, and the all-age mortality rate from CKD increased 41.5% worldwide between 1990 and 2017 (GBD Chronic Kidney Disease Collaboration, 2020). Meanwhile, the global all-age prevalence of CKD increased 29.3% since 1990; however, the age-standardized prevalence remained stable (1.2%, −1.1–3.5) (GBD Chronic Kidney Disease Collaboration, 2020). Compared with other non-infectious diseases, the global age-standardized mortality and prevalence rates for CKD are not declining, which indicates that increasing investment in diagnosis technology and targeted drug development, as well as establishing sustainable healthcare infrastructure for CKD, is a global priority (Okpechi et al., 2017).

CKD is generally characterized by glomerular filtration rate (GFR) less than 60 ml/min/per 1.73 m^2^ or albuminuria 30 mg per 24 h and the existence of urinary and serum biomarkers (Romagnani et al., 2017; Webster et al., 2017). Combined with clinical manifestations, renal histopathology and albuminuria, estimated glomerular filtration rate (eGFR), and the common index of renal function are normally used instead of GFR to diagnose CKD based on serum creatinine (Scr) level in clinical practice (Romagnani et al., 2017; Webster et al., 2017). However, the obvious limitation with the diagnostic criteria is the concentration of Scr, which varies greatly based on gender, age, muscle mass, muscle metabolism, overall body weight, hydration status, and nutritional status, causing the changes in Scr level to be not synchronized with the decreased renal function (Goek et al., 2012; Ferguson et al., 2015). Meanwhile, the non-renal processes such as fever and infection could affect the assessment of proteinuria (Couser, 2017; Luis-Lima and Porrini, 2017). Furthermore, proteinuria may persist some time after healing primary renal diseases, leading to unnecessary treatment for patients with renal diseases (Touma et al., 2014). The limitations mentioned above could subsequently impair clinicians’ abilities to accurately identify individuals at the risk and prognosis of CKD when treatments may mitigate their future risks as recommended in guidelines (Wouters et al., 2015). Therefore, under high potential for mislabeling CKD status based on eGFR and proteinuria, it is urgent and beneficial to investigate sensitive diagnostic measures and novel biomarkers, which are receiving increasing attention to improve the diagnostic and prognostic efficiency and surveillance of CKD (Rysz et al., 2017).

With the development of genomics (Vivante and Hildebrandt, 2016; Graham et al., 2019; Morris et al., 2019; Wuttke et al., 2019), transcriptomics (Lake et al., 2019), proteomics (Cañadas-Garre et al., 2019; Dubin and Rhee, 2020; Verbeke et al., 2021), and metabolomics (Hocher and Adamski, 2017; Gagnebin et al., 2018; Kalantari and Nafar, 2019), the high-throughput and high-resolution multi-omics interactive analysis strategy and machine learning data mining would be beneficial to investigate novel biomarkers for further understanding CKD progression and prognosis, thereby advancing and improving therapeutics (Davies, 2018). Owing to the increasing prevalence of diabetes mellitus, hypertension, and environmental pollution (Liu B et al., 2020) (exposed to PM 2.5 and PM 10), the cause of CKD is complex and uncertain and the progression of CKD (5 stages based on eGFR or albuminuria) is multivariate that ultimately leads to different CKD prognosis. The progression of CKD could be alleviated when CKD was predicted or diagnosed in an early stage. Unfortunately, the absence of sensitive and specific biomarkers further complicates the early prediction and diagnosis. Meanwhile, the healthcare interventions aiming to delay the progression of CKD and prevent negative outcomes are extremely limited (Wang Y. N et al., 2019). As such, discovering novel biomarkers is urgently needed for clinicians and researchers to have in-depth knowledge of the highly interconnecting genetic and molecular networks; then, the illumination of underlying mechanisms could be conducive to assessing the risk of CKD as early as possible, developing precise therapeutic strategies in progression, and accurately evaluating the prognosis.

In this review, we highlighted the latest-reported biomarkers, which incorporated progression monitoring and prognosis of CKD. The specificity and functional correlation of these biomarkers were discussed from the perspective of the mechanism and clinical significance to provide a basis for the further development of these biomarkers, the optimization of CKD precise diagnosis and treatment schemes, and the targeted medicine development.

## Progression Biomarkers of CKD

The pathogenesis of CKD progression is complex, which is independent of the primary cause and leads to irreversible nephron loss, ESRD, and death. It had been well-investigated that parenchymal cell loss, chronic inflammation, fibrosis, and reduced regenerative capacity of the kidney were the major factors contributing to CKD progression. Exploring biomarkers that could predict or monitor the progression of CKD is a hot issue in current CKD research.

### Renal Fibrosis

As the most important pathological manifestation in the progression of CKD, renal fibrosis is part of the normal repair process that is triggered in response to injury and preserves the architecture and functional integrity of the tissue. However, deregulation of this process is a decisive factor leading to renal failure. After the renal injury, pro-fibrosis factors such as connective tissue growth factor (CTGF) (Yin and Liu, 2019), transforming growth factor-β (TGF-β) (Miao et al., 2021a), sonic hedgehog (Shh) (Ren et al., 2020), and Wnts (Hu et al., 2020) would be secreted by renal tubular epithelial cells, intrinsic renal cells, macrophages, lymphocytes, and other immune cells. These fibrogenic factors would trigger transdifferentiation of various renal cells into myofibroblasts, induce epithelial-to-mesenchymal Transition (EMT), and promote the stimulation of extracellular matrix (ECM) (Liu et al., 2018). In the early stage of renal injury, the deposition of the fibrotic matrix contributes to the repair of renal tissue before being absorbed by repaired tissue. However, during the persistent injury of CKD, the fibrotic matrix would be overdeposited (Ma and Meng, 2019). Excessive ECM decreased the degradation rate of ECM-degrading enzymes such as matrix metalloproteinases (MMPs) and increased the deposition of ECM. Damaged cells continued to secrete fibrogenic factors and aggravated the process of fibrosis. These pathological changes lead to the destruction of tissue structure, the decrease of renal function and the ability of tissue repair, and finally renal failure (Humphreys, 2018). Exploring the biomarkers reflecting renal fibrosis could evaluate subtle progression, estimate prognosis, and facilitate clinical management of CKD individuals timely and accurately.

#### MMPs

MMPs, a large family of endopeptidases, could affect CKD progression through reshaping and degrading cellular ECM components and regulating non-ECM molecules that play an important role in the development of CKD, including adhesion molecules, cytokines, and growth factors, under the strict regulatory mechanisms (Lindsey et al., 2016). Overall, It had been found that 11 members (MMP-1, MMP-2, MMP-3, MMP-7, MMP-9, MMP-13, MMP-14, MMP-24, MMP-25, MMP-27, and MMP-28) are expressed in the kidney.

Among them, MMP-9 plays the most representative role in the progression of renal fibrosis. The expression of MMP-9 is low in a normal physiological state but active in persistent renal injury. In the experimental model of renal fibrosis, the expression of MMP-9 was found in mesangial cells, glomerular cells, epithelial cells and endothelial cells, fibroblasts, macrophages, and neutrophils (Tan and Liu, 2012). Furthermore, inhibition of MMP-9 could reduce the infiltration of neutrophils and other inflammatory cells and reduce the degree of renal fibrosis (Wang H et al., 2019). MMP-9 was also abnormally expressed in CKD patients. Urinary MMP-9 levels were significantly higher in children with CKD than those in age-matched healthy controls (Musiał et al., 2015) and were increased in patients with focal segmental glomerulosclerosis (Korzeniecka-Kozerska et al., 2013). In addition, a cohort study involving 251 adults showed that the increase in plasma MMP-9 could predict the progression of CKD after 8.5 years with a risk ratio of 4.7 (Hsu et al., 2013). Above all, current evidence supported the potential of MMP-9 as a biomarker of CKD progression.

In addition to MMP-9, MMP-2 also shows a high correlation with CKD progression. Nadkarni GN et al. found that the increase of MMP-2 per unit of urine led to a decrease in eGFR (0.1 ml/min/m^2^) during the 38-month follow-up (AUC 0.74) (Nadkarni et al., 2016). Such results were also confirmed by another 8-year follow-up study on patients after coronary angiography: patients with higher levels of MMP-2 are 2.5 times more likely to have a decline in eGFR (Hsu et al., 2013). Moreover, a systematic review showed that MMP-2 has the potential to identify patients at risk of renal fibrosis that leads to worse renal outcomes (Mansour et al., 2017). However, more evidence is still needed to determine the role of MMP-2 in evaluating the progression of renal fibrosis in CKD.

MMP-7 is a secreted zinc- and calcium-dependent endopeptidase and a transcriptional target of canonical Wnt/β-catenin signaling. It had been demonstrated that Wnt/β-catenin could be activated in kidney disease and the level of urinary MMP-7 might be used as a noninvasive surrogate biomarker (Liu Z et al., 2020) and a therapeutic target for renal fibrosis (Wozniak et al., 2021). Zhou et al. (2017) conducted a cross-sectional study under measuring urinary MMP-7 levels in a cohort of 102 patients with CKD compared with normal subjects. It indicated that urinary MMP-7 levels, elevated in kidney disorder patients, closely correlated with renal fibrosis scores. Meanwhile, the knockout of MMP-7 could ameliorate fibrotic lesions and matrix gene expression in mice. The expression of E-cadherin protein could be preserved by the genetic ablation of MMP-7 and substantially reduced the expression of total and dephosphorylated β-catenin. Li et al. (2021) reviewed that MMP-7 could activate the Wnt/β-catenin signaling pathway after renal injury and urinary MMP-7 may be a noninvasive biomarker of profibrotic signaling in the kidney. In contrast, they discovered that MMP-7 exerts protective effects on the kidney as an adaptive response in cisplatin administration or folic acid-induced AKI animal models. Therefore, further study should be performed to explore the role of MMP-7 as a therapeutic target of renal fibrosis.

#### Monocyte Chemotactic Protein 1

MCP-1 belongs to the chemokine family and has a strong chemotactic effect on monocytes (Das et al., 2021). This unique cytokine is mainly produced by chemokine in renal intrinsic cells and macrophages in response to hypercholesterolemia and other arterial injuries (Gregg et al., 2017; Tam and Ong, 2020). MCP-1 plays an important role in developing renal inflammation and fibrosis under the stimulation of oxidative stress, cytokines, or growth factors.

It had been reported that MCP-1 was deeply implicated in albuminuria and kidney dysfunction. The upregulation of MCP-1 was accompanied by the activation of IκB/NF-κB signaling in CKD patients with macroalbuminuria; the similarity result had also been found that the activation of NF-κB was accompanied by significant upregulation of MCP-1 in CKD rats (Feng et al., 2019). The researchers found in animal models that blocking MCP-1 receptors might suppress inflammation and alleviate glomerulonephritis (Khalili et al., 2020). In addition, the potential of MCP-1 as a biomarker of renal fibrosis has also been confirmed in clinical trials. In a cohort study of 58 patients with IgA nephropathy, urinary MCP-1 levels provided significant additional non-invasive information for a better predictive performance of the severity of interstitial fibrosis beyond traditional markers (Segarra-Medrano et al., 2017). Recently, a probabilistic sampling longitudinal cohort study involving 3,257 participants was found that the MCP-1 of CKD patients was higher than that of non-CKD participants, and the level of MCP-1 was negatively correlated with eGFR. Furthermore, this study also proved that a high level of MCP-1 was an independent risk factor for CKD death (Gregg et al., 2018). Nadkarni GN et al. found that the ratio of urinary MCP-1 to creatinine was associated with a sustained decline of more than 40% in eGFR and showed better risk predictions than other traditional indicators (Tam and Ong, 2020). Besides, a case-control study also found that the level of MCP-1 was positively correlated with the probability of CKD (Zhang et al., 2018). Another sensitivity analysis involving 171 matched cases and controls suggested that the ratio of urinary MCP-1 to creatinine was correlated with the incidence of CKD stages (Musiał and Zwolińska, 2020). It indicated that MCP-1 is of high predictive value in evaluating the progression and prognosis of renal fibrosis in CKD.

#### Dickkopf-3

DKK-3, a member of the glycoprotein family (DKK1-4) (Gröne et al., 2017), is a recently discovered stress-induced tubular-derived renal biomarker for interstitial fibrosis (Federico et al., 2016; Fang et al., 2020; Seibert et al., 2021). It mainly promotes renal tubular epithelial cells producing fibrogenic molecules secreted by stress stimulation (Schunk et al., 2021a). Expression of DKK-3 in the developing kidney would cease after kidney maturation and would reignite when the kidney tissue is damaged (Seibert et al., 2021). Lipphardt et al. (2019) have found that DKK3 could significantly reduce the area percentage, total length, and bifurcation number of capillary structures in renal microvascular endothelial cells. Furthermore, it indicated that the nephropathy induced by adriamycin could lead to the upregulation of the DKK-3 level in renal tubular. As a result, DKK-3 could induce EMT and impair angiogenic competence, giving rise to renal fibrosis. In addition, DKK-3 could stimulate the expression of TGF-β, which is recognized as an important factor leading to renal fibrosis (Karamariti et al., 2018).

In the animal model of CKD, DKK-3 could regulate the signal transduction of the Wnt/β-catenin signal pathway and induce renal tubulointerstitial fibrosis (Schunk et al., 2021b). Another cohort study showed that increased urinary DKK-3 concentration in patients with primary glomerular disease and primary interstitial disease was significantly associated with higher levels of tubulointerstitial fibrosis, and the significant eGFR decline within 6 months is closely related to the level of urinary DKK-3 (Zewinger et al., 2018). The results suggested that DKK-3 might be a sensitive predictor in the early progress of CKD.

#### 5-Methoxytryptophan

5-MTP, converted by TPH-131, is an endogenous tryptophan metabolite that could reduce the production of inflammatory cytokines and thus ameliorate inflammation and tissue damage by inhibiting NF-κB activation and the consequent inhibition of COX-2 transcriptional activation.

In recent research, Chen D. Q et al. (2019) identified 5-MTP for the first time as the most promising biomarker metabolite for detecting early CKD. An untargeted metabolomics study of 2,155 participants, including patients with stage 1–5 CKD and healthy controls, was performed, and the level of 5-MTP decreased with the progression of CKD that strongly correlates with clinical markers of kidney disease. Furthermore, the biological effects of 5-MTP were performed using UUO mice and HK-2 and HMC cells. It indicated that 5-MTP attenuated the expression of the pro-inflammatory factor NF-κB p65 and its target gene products MCP-1 and COX-2 while increasing the expression of the anti-inflammatory and antioxidant transcription factor Nrf 2 and its target gene products HO-1 and NQO-1. In addition, 5-MTP treatment could significantly attenuate the upregulation of the pro-fibrotic proteins collagen I, fibronectin, wave protein and α-SMA and reverse the downregulation of E-cadherin and Thy1 *in vivo* and *in vitro* settings. The present study demonstrated that 5-MTP could separate patients with early stage CKD from healthy controls and serve as potential biomarkers of the early stage of CKD.

#### 1-Aminopyrene and 1-Hydroxypyrene

A recent study have discovered that aryl-containing metabolites might have an important effect on chronic kidney disease progression. Most importantly, two polycyclic aromatic hydrocarbon metabolites, 1-aminopyrene (1-AP) and 1-hydroxypyrene (1-HP), showed strong positive and negative correlation with serum creatinine and creatinine clearance, respectively. Miao et al. (2020), Miao et al. (2021b) analyzed 5,406 urine and serum samples from patients with stage 1–5 CKD using metabolomics; 1-AP and 1-HP were identified and validated using longitudinal and drug intervention cohorts and 5/6 nephrectomized and adenine-induced rats. Furthermore, the increased levels of 1-AP and 1-HP in serum and kidney tissues correlated with decreased renal function in two rat models. Upregulated mRNA expression of aryl hydrocarbon receptor and its target genes, including CYP1A1, CYP1A2, and CYP1B1, were observed in patients and rats with progressive CKD. It indicated that 1-AP and 1-HP were demonstrated to mediate renal fibrosis through activation of the aryl hydrocarbon receptor signaling pathway and the endogenous 1-AP and 1-HP are novel mediators of CKD progression.

### Inflammatory States

Inflammatory states, a common pathological change in all stages of CKD progression, are essential in renal fibrosis and renal loss and are closely associated with CKD complications (Akchurin and Kaskel, 2015). The factors contributing to the inflammatory states of CKD might include the increased production of pro-inflammatory cytokines, acidosis, oxidative stress, recurrent infections, and intestinal malnutrition.

Comparatively, inflammatory cytokines are the key to the micro-inflammatory state of CKD. Inflammatory cytokines, secreted by circulating monocytes and endothelial cells, could induce micro-inflammatory states through the bloodstream. The kidney receives 25% blood volume of the body and yet lacks defense against inflammation and oxidative stress. Therefore, the kidney becomes one of the organs most susceptible to microinflammation (Mihai et al., 2018). Circulating proinflammatory cytokines would activate renal micro-vessels, especially endothelial cells and leukocytes, and lead to local amplification of proinflammatory factors and reactive oxygen species. These processes could affect adhesion molecules on the cell surface and impair endothelial barrier function, activation of the coagulation system, and receptor-mediated vascular reactivity. The above inflammation-mediated changes may lead to irreversible tubular damage and renal unit failure (Qian, 2017). Therefore, using biomarkers such as proinflammatory cytokines to understand the inflammatory state of CKD is not only of great significance for evaluating the progress of CKD and judging the prognosis but also providing guidance for improving the inflammatory state of CKD through clinical intervention measures such as diet, medicine, and dialysis (Akchurin and Kaskel, 2015).

#### Common Biomarkers of Inflammation

Multiple evidence have shown that the commonly inflammatory indicators, such as IL-1, IL-6, C-reactive protein (CRP), and tumor necrosis factor (TNF)-like weak inducer of apoptosis (TWEAK), could effectively judge the progression and prognosis of CKD. A study showed that patients with chronic kidney disease with lower eGFR levels had higher levels of plasma IL-1 β, IL-1RA, IL-6, TNF-α, hypersensitive CRP, and fibrinogen and that the inflammation score was inverted with GFR estimates and urinary albumin/creatinine ratio (UACR) (Gupta et al., 2012). A recent cohort study involving 3430 patients with chronic renal insufficiency also found that increased levels of TNF-α and decreased serum albumin in patients with chronic renal disease were associated with rapid loss of renal function (Amdur et al., 2016). Studies also have shown that TEWAK worked as an important factor in inflammation-related renal injury and accelerates the progression of CKD (Sanz et al., 2014).

#### Inflammasome

The inflammasome is a large polyprotein complex induced by lipopolysaccharide (LPS). It had been a hot spot in the field of nephrology recently and was first reported as an innate immune signaling pathway in 2002 (Martinon et al., 2002).

Intracellular NOD-like receptors (NLRs) play a key role in forming and activating inflammatory bodies. They could form the inflammatory complex and release proinflammatory cytokines IL-1 β and IL-18 (Akchurin and Kaskel, 2015; Hutton et al., 2016). NLRP3 inflammasome is closely related to apoptosis, autophagy, and proinflammatory cytokines regardless of traditional microbial innate immune stimulation; therefore, it is of extensive significance for a variety of renal diseases (Chang et al., 2014).

The role of NLRP3 in the formation of the inflammatory state of CKD had been confirmed in animal experiments. In the model of unilateral ureteral obstruction, the renal tubular injury, inflammation, and fibrosis were less severe in NLRP3-knockout mice (Vilaysane et al., 2010). In addition, a bidirectional Mendelian randomization (MR) analysis showed that elevated NLRP3 inflammasome had a profound impact on the pathogenesis and severity of CKD (Jia et al., 2019). A clinical cohort study also showed that the expression of NLRP3 mRNA in CKD patients was significantly upregulated compared with that of the control group (El-Deeb et al., 2019). However, although NLRP3 has the potential to be used as a biomarker to evaluate the progression of CKD, future research is needed to provide solid evidence to evaluate its diagnostic performance.

### Disorder of Gut–Kidney Axis

Since Ritz proposed the concept of “intestinal syndrome” at the International Dialysis Conference in 2011 (Ritz, 2011), the connection between the intestine and the kidney has been extensively studied, and the “gut-renal axis” theory has been derived. It is believed that the connection between the “gut-renal axis” is two-way, indicating if the function of the gut is damaged, the normal function of the kidney could be affected in various ways and vice versa. Gut microbiota and their metabolites have been investigated that could play significant roles in the “two-way affection.” The disruption of gut microbiota may lead to intestinal dysbiosis, intestinal barrier dysfunction, and bacterial translocation; then indoxyl sulfate (IS), p‐cresyl sulfate (PCS), and trimethylamine‐N‐oxide (TMAO) were produced as a result of gut microbiota alteration, which could be implicated in the variant processes of kidney diseases development (Chen L et al., 2019).

Recently, more evidence has shown that the crosstalk in the “gut–kidney axis” is closely related to CKD progression. The reduction in renal filtration capacity occurs commonly in renal failure patients, leading to the accumulation of urea in the blood. After urea diffuses into the intestinal tract, it will destroy the tightly connected proteins of the colon, increase the permeability of the intestinal tract (Vaziri et al., 2013), and lead to a flora imbalance characterized by the proliferation of facultative anaerobes (Wuttke et al., 2019; Litvak et al., 2018). The increased intestinal permeability could cause bacteria and their metabolites to enter the blood from the intestinal cavity, accompanied by systemic inflammation. The disorders of intestinal microflora (such as species richness, diversity, composition, and function) change the selection of nutrients and bioactive metabolites, resulting in the accumulation of intestinal uremic toxins, the increase in the circulation LPS levels, and the impairment of immune function, which are considered as the key factors in the occurrence and development of CKD and its complications (Le Chatelier et al., 2013).

#### Indoxyl Sulfate

Tryptophan metabolites from gut microbiota have been demonstrated to participate in renal fibrosis as aryl hydrocarbon receptor ligands. Indole derivatives are derived from tryptophan metabolism pathways modulated by gut microbiota directly or indirectly. IS is a metabolite of indole produced by tryptophan through the intestinal flora (including *E. coli*) (Evenepoel et al., 2009). IS could activate the renin-angiotensin-aldosterone (RAS) system (Sun et al., 2012), induce cell senescence and apoptosis (Han et al., 2018), and promote EMT (Kim et al., 2012), thus accelerating the progression of fibrosis, renal dysfunction, and CKD.

A variety of trials had confirmed the value of IS in predicting CKD. A study showed that at the earliest stage of CKD, the change of IS is proportional to the severity of the disease (Kim et al., 2020). Serum IS levels in CKD patients were directly related to dialysis events; therefore, IS might potentially predict the progression of advanced CKD (Wu et al., 2011). Moreover, a recent cohort study involving 100 CKD patients and 30 patients with normal renal function showed that the level of IS reflected the degree of renal impairment and peaked in the late stage of CKD (Wu et al., 2020). IS could be used as a novel biomarker for a thorough evaluation of CKD progression and future research is needed to provide solid evidence to evaluate its diagnostic function.

#### P-Cresyl Sulfate

PCS is produced through the transformation of cresol by intestinal epithelial cells. PCS induces renal injury and fibrosis by inhibiting *klotho* gene expression, activating the RAS/TGF-β pathway (Sun et al., 2012), inducing EMT, and causing NADPH oxidase-driven ROS (Watanabe et al., 2013). A prospective observational trial involving a cohort of 268 patients with specific CKD stages showed that serum total PCS was independently associated with renal progression (Barrios et al., 2015). Another cohort study of 4,439 individuals with different eGFR also demonstrated that PCS was negatively correlated with eGFR and PCS might gradually increase along the early process of CKD (Chen L et al., 2019). Furthermore, several studies have also shown that PCS levels in patients with CKD are associated with poor prognosis, including death (Shafi et al., 2015; Lu et al., 2016). Since the change of PCS might be sensitive to the slight process of early CKD, it could be a valuable predictor for CKD progression.

#### Trimethylamine N-Oxide

TMAO, one of the major uremic toxins, is byproduct of bacterial metabolism of phosphatidylcholine, choline, or L-carnitine. It had been confirmed to be elevated in patients with CKD and closely associated with a decreased renal function (Tang et al., 2015). Chen et al. investigated that both plasma and urine TMAO were significantly elevated in 180 CKD patients compared to those of 120 age-matched healthy controls patients by determining their fasting plasma and urine samples by UPLC-HDMS-metabolomics and quantitative real-time RT-PCR techniques. The concentration of TMAO in CKD patients plasma had been shown to directly correlate with plasma concentration of urea and creatinine, indicating the close association of TMAO with the degree of renal insufficiency (Chen et al., 2017).

TMAO might induce oxidative stress by inhibiting the expression of the oxidative stress inhibitor SIRT1, increasing H_2_O_2_, and reducing SOD activity (Ke et al., 2018). TMAO could affect systemic inflammatory response (Missailidis et al., 2016) by regulating inflammatory inducers (IL-6, TNF-α, and so on), promoting the phosphorylated NF-κB to enter the nucleus (Seldin et al., 2016), and activating the expression of inflammasomes (Boini et al., 2017). In clinical practice, a cohort study of 80 patients at all stages of CKD observed significant increases in urinary and plasma TMAO levels in the CKD group and significantly higher levels in stage 4 CKD (Wu et al., 2020). Consistent results have been reported in an experiment that TMAO level was negatively correlated with renal function decline and returned to normal after kidney transplantation (Missailidis et al., 2016). Furthermore, TMAO was an independent predictor of mortality in patients with CKD.

#### Gut Microbiota

Microflora refers to all the coexisting microorganisms living in the host, which are mainly composed of bacteria, viruses, archaea, fungi, and unicellular eukaryotes (Evenepoel et al., 2017; Castillo-Rodriguez et al., 2018; Cosola et al., 2018). It has been estimated that there are at least 35,000 different microorganisms in the human body (Frank et al., 2007). Under normal circumstances, the intestinal microflora is mainly composed of five phyla, among which Firmicutes and Bacteroidetes account for the largest proportion in the colon, followed by Actinobacteria, Verrucomicrobia, and Proteobacteria (Vaziri, 2012).

The microflora is responsible for metabolizing dietary components and improving digestion by expressing several enzymes (Plata et al., 2019). The microflora, with its metabolic activity, could affect the health of the host by interfering with the physiology, nutrition, metabolism, and immune system (Le Chatelier et al., 2013)^;^ (Human Microbiome Project Consortium, 2012). Researchers have investigated that obesity, type 2 diabetes (T2DM), and cardiovascular diseases (CVD) might be associated with complex interactions between gut microflora and human hosts (Gurung et al., 2020; Zhou et al., 2020).

Emerging evidence also suggested that intestinal microflora plays an important role in the occurrence and development of CKD (Vanholder and Glorieux, 2015; Nallu et al., 2017). Therefore, the changes of intestinal microflora might provide a possibility for measuring the progression of CKD. Barrios et al. found that the changes in Ruminococcaceae, Christensenellaceae, and Lachnospiraceae families were significantly correlated with eGFR at the early stage of CKD (Barrios et al., 2015). Furthermore, Wu and his colleagues found that accessory *Escherichia coli* (AUC 0.78; 95% CI 0.7–0.87) and pseudobutyric acid bacillus (AUC 0.76; 95% CI 0.67–0.84) could effectively distinguish CKD from the health control compared to urinary protein/creatinine ratio and sterins *Collinsella stercoris* has shown excellent performance in identifying healthy people and early CKD patients (Wu et al., 2020). This indicated that microflora could be a valuable tool to evaluate the progress of CKD, especially in the early stage.

## Biomarkers for Early Diagnosis of CKD Complications

The progression of CKD is associated with various complications, which have a higher prevalence and intensity with the decrease of renal function. CKD complications could result in high morbidity and mortality that reduce the quality of life. It has been reported that CVD, hypertension, anemia, mineral bone disorders, volume overload, electrolyte, and acid-base abnormalities are common complications of CKD. The high morbidity and mortality of CKD complications have brought a considerable burden on global medical resources. A better understanding of CKD-related complications might help optimize the diagnosis, prevention, and management of CKD.

### Cardiovascular Disease Based on CKD

CVD is a common complication in CKD patients, which is negatively asssociated with the renal function (Herzog et al., 2011). According to the American kidney data system report published in 2013, for CVD patients, co-occurrence of CKD might increase the risk of heart failure from 18.5 to 43% and the risk of acute myocardial infarction from 6.4 to 15% (Diez Roux et al., 2016). In addition, a cohort study based on prospective populations found that early CKD patients without vascular disease were associated with a subsequent risk of coronary heart disease (Briasoulis and Bakris, 2013; Zhang et al., 2019). It has been found that risk factors attributed to impaired renal function, such as inflammation, oxidative stress, hyperphosphatemia, hypercalcemia, and secondary hyperparathyroidism, could increase cardiovascular risk in patients with renal disease (Frank et al., 2007).

Thus, accurate assessment of cardiovascular risk is critical to the daily treatment decisions of patients with CKD. However, conventional markers of myocardial injury, such as troponin (Yeh Michoset al., 2014) or brain natriuretic peptide (Takase and Dohi, 2014), may generally be chronically elevated due to the decrease of clear renal function, which cannot accurately assess the risk of cardiovascular events in patients with CKD. Therefore, it is urgent to find more accurate new biomarkers. Currently, several biomarkers have been found with great potential.

#### Galectin-3

As a beta-galactoside binding protein, Gal-3 is ubiquitous in cells and it could be secreted into the extracellular space by epithelial cells, endothelial cells, and macrophages (Wang and Guo, 2016) in the kidney and heart. It had been indicated that Gal-3 could play an important role in initiating cardiac fibrosis and ventricular remodeling (Gleissner et al., 2017). Gal-3 could modulate kidneys pro-inflammatory effects, regulate growth, differentiation, proliferation of the cells and moreover mediate aldosterone-induced fibrosis of the heart and blood vessels (Calvier et al., 2013; Madrigal-Matute et al., 2014; Vergaro et al., 2016). Furthermore, researchers have found that Gal-3 is also highly expressed in injured kidneys and is involved in the progression of renal fibrosis (Nikolic-Paterson et al., 20112014). O’Seaghdha CM et al. found that higher plasma Gal-3 levels were associated with a rapid decline in eGFR based on an analysis of kidneys from 2,450 patients who were followed for an average of 10 years (O’Seaghdha et al., 2013). Considering its important role in the progression of both cardiac and renal fibrosis, Gal-3 has the potential to predict the risk and prognosis of CVD in patients with CKD.

A postmortem analysis of a four-dimensional study of 1,168 diabetic HD patients showed that circulating Gal-3 levels were negatively correlated with renal function and were four to five times above the reference range; meanwhile, Gal-3 was associated with cardiovascular events (Drechsler et al., 2015). Studies with 5,226 patients have confirmed that the level of Gal-3 increased by 1% and the risk of all-cause death increased by 37.9% in CKD patients (Zhang et al., 2019). In summary, Gal-3 could be an effective biomarker for predicting cardiovascular events in CKD.

#### Soluble ST-2

As a member of the IL-1 receptor family, ST-2 contains two forms as soluble ST-2 (sST-2) and transmembrane ST-2 (ST-2L). Elevated sST-2 might be highly associated with adverse outcomes in patients with CVD, acute and chronic heart failure, or even death (Dieplinger et al., 2014; Savic-Radojevic et al., 2017). It has been approved by FDA as a new type of biomarker for clinical use to evaluate risk stratification and treatment guidance for patients with acute and chronic heart failure (Aimo et al., 2017; Dalal et al., 2018).

In recent years, emerging evidence suggested its potential role as a biomarker for predicting cardiovascular events in CKD. A study confirmed that heart failure patients with elevated sST-2 and eGFR<60 ml/min/1.73 m^2^ had a significantly increased risk of death (Bayes-Genis et al., 2013). A head-to-head comparison study with Gal-3 in patients with chronic heart failure also demonstrated that sST-2 performed better than Gal-3 and could predict 5-year risk of cardiovascular death (Bayes-Genis et al., 2014). The significant correlation between plasma sST-2 levels and the progression of CKD to ESRD was also confirmed in a recent cohort study involving 219 patients who participated in the German Chronic Kidney Disease (GCKD) study (Mirna et al., 2020). All the evidence demonstrated the importance of sST-2 in evaluating the cardiovascular risk of CKD.

#### Growth Differentiation Factor-15

As a member of the TGF-β cytokine family, GDF-15 is widespread in mammalian tissues, including the prostate, intestinal mucosa, and kidney (Unsicker et al., 2013). It has been found to be involved in cancer, obesity, and cardiovascular and kidney diseases (Nair et al., 2017). Previous studies suggested that GDF-15 might take parts in tissue inflammation, oxidative stress, and injured cardiomyocyte repair and show anti-apoptosis and anti-hypertrophy effects (Kempf et al., 2006).

Several clinical studies have found that higher levels of GDF-15 are associated with poor prognosis, including CVD, heart failure, and death in patients with CKD (Daniels et al., 2011; Breit et al., 2012; Lindman et al., 2015; Bansal et al., 2019). Meanwhile, GDF-15 is associated with CKD events and rapid decline in renal function. Adding GDF-15 to clinical covariates could also improve the prediction of CKD events (Ho et al., 2013). These studies confirmed that the increase of GDF-15 may be a physiological signal of early heart failure, and GDF-15 is helpful in evaluating the risk of CVD in patients with CKD.

### Diabetic Nephropathy

Diabetic nephropathy (DN) could occur in approximately 30–40% of patients with diabetes and also account for the major cause of CKD (Tesch, 2017; Qi et al., 2018; Umanath and Lewis, 2018). Hyperfiltration and albuminuria in the early stage are the typical presentation of DN, followed by a progressive renal function decline and eventually progressed to CKD (Sagoo and Gnudi, 2020). Early diagnosis may prompt interventions and improve prognosis. Recent researches have reported that traditional risk factors such as proteinuria could not effectively predict DN progression, particularly in GFR decliners without increased albuminuria (Yamamoto et al., 2018). Consequently, delayed initiation of clinical therapy might preclude adequate prevention of progression to ESRD (Yamamoto et al., 2018).

Developing novel biomarkers is essential not only for diagnosing high-risk patients and predicting disease progression at the incipient stage but also for identifying new players in the pathogenesis of glomerular injury in diabetes. Currently, several biomarkers have been extensively investigated for predictive performance on early prediction and diagnosis of DN (Gluhovschi et al., 2016).

#### CypA

CypA, a cytoplasmic protein, has isomerase activity and could catalyze the trans-to-cis change of the peptide bond on the proline residue (Hoffmann and Schiene-Fischer, 2014). CypA could act as inflammatory mediators under the stimulation of oxidative stress, inflammation, and hypoxia and participate in the process of inflammation and apoptosis by affecting multiple processes of transcriptional signal transduction (Sherry et al., 1992; Kim et al., 2004; Suzuki et al., 2006). In the kidney, CypA exists mainly in proximal tubules and renal tubular injury has been proved to be the leading cause of proteinuria and hyperfiltration of renal tissue in diabetic nephropathy (Demeule et al., 2000; Zeni et al., 2017). Studies found that plasma monocytes in patients with diabetes secrete a large amount of CypA in response to hyperglycemia, suggesting that CypA may be a potential secretory marker for T2DM (Ramachandran et al., 2012). Therefore, it could be detected in urine or plasma of patients with diabetes and may increase in patients with DN.

In 2015, a cross-sectional study found the connection between the concentration of urinary CypA and the progression of renal function with good discriminatory power (sensitivity of 90.0%, specificity of 72.7%, AUCs = 0.85) for diagnosing stage 2 DN; therefore, the authors proposed that urinary CypA could act as a new biomarker for early DN (Tsai et al., 2015). A cross-sectional and longitudinal study showed that baseline plasma CypA was positively correlated with changes in glomerular filtration rate for patients with T2DM regardless of the cutoff level or persistent level and could serve as indicators of renal disease progression in T2DM patients (Chiu et al., 2018). In addition, another cross-sectional study of 137 patients with T2DM also verified that CypA has high accuracy in the early diagnosis of DN: AUCs of CypA were 0.914 and 0.937, respectively, for the prediction of incipient and overt DN (Abdel Ghafar et al., 2020).

#### Periostin

Periostin is an original member of the stromal cell protein family (Frangogiannis, 2012). When binding to αVβ3-and α Vβ5-integrins, periostin could activate a scaffold protein complex with adaptor proteins and α-parvin (Wallace, 2019). In normal tissue, ILK-PINCH-parvin (IPP) activates EMT and stimulates the cellular signal pathway involved in tissue repair, thus protecting the integrity of the extracellular matrix (Cobo et al., 2016).

EMT is one of the key mechanisms involved in DN development (Loeffler and Wolf, 2015); during EMT, increasing mesenchymal biomarkers like periostin is very characteristic (Conway et al., 2014). Previous studies have shown that periostin could increase the expression of TGF-β, one of the most important fibrotic factors that could directly promote EMT and stimulate ECM synthesis, and therefore induce extracellular matrix deposition (Gordon et al., 2012). Interestingly, renal fibrosis and inflammatory mediators, including TGF-β, angiotensin II, PDGF-B, and IL-4 and IL-13, could upregulate the expression of periostin (Olsan et al., 2018). The study also showed that the administration of TGF-β to adrenal epithelial cells could increase the expression of periostin and stimulate the transformation of renal epithelial cells (Mael-Ainin et al., 2014). Therefore, it is reasonable to believe that the positive feedback regulation between periostin and TGF-β may play a key role in EMT, and abnormal overexpression of periostin would lead to glomerulonephritis and interstitial fibrosis and cause persistent damage to the kidney in DN.

Recent studies have found that periostin could be an effective tissue biomarker for predicting renal damage in patients with DN. Periostin is not expressed in healthy adult kidneys (Satirapoj et al., 2015); however, renal tubular epithelial cells would secrete a high level of periostin to the renal tubule interstitial in response to different severity of renal injury (Sen et al., 2011; Satirapoj et al., 2012). Both experimental and clinical studies showed that periostin might act as a key biomarker of DN. Increased expression of periostin was positively related to the severity of renal fibrosis in the mouse model of bilateral kidney and unilateral ureteral obstruction (Um et al., 2017). In addition, a significant increase in periostin was also found in the kidneys of streptozotocin-induced diabetic mice after nephrectomy (Satirapoj et al., 2012). An observational study involving 19 healthy controls and 71 DN patients found a strong positive correlation between urinary albumin-creatinine ratio (UACR) and urinary periostin levels, and the AUC of periostin for the diagnosis of established microalbuminuria was 0.833, indicating that periostin could be considered as reliable biomarkers in the diagnosis of DN (El-Dawla et al., 2019). The study also found that the level of urinary periostin was significantly higher than the normoalbuminuria group, with the AUCs of periostin being 0.954 and 0.997, respectively, for the prediction of DN (Abdel Ghafar et al., 2020). In addition, it has been found that the level of urinary periostin was gradually increased for healthy controls and T2DM patients with normal albuminuria, microalbuminuria, and massive albuminuria; therefore, the increased level of urine periostin could be detected in T2DM patients earlier than significant albuminuria (Satirapoj et al., 2015). Therefore, periostin could be regarded as a valuable urinary biomarker for predicting early DN in patients with T2DM.

#### MicroRNAs

MicroRNAs are a group of non-coding RNA composed of 19–24 nucleotides (Zhou et al., 2014). miRNAs could cleave mRNA or suppress the translation by interacting with the complementary sequence in the 3′-untranslated region of its mRNA target, followed by regulating gene expression (He and Hannon, 2004). They contributed to the physiological processes of cell proliferation, differentiation, and death (Sayed and Abdellatif, 2011). The specific expression of miRNA has been found in the development of many diseases, such as cancer and chronic lymphoblastic leukemia (Khan et al., 2019; Javandoost et al., 2020). Recent studies have found that the abnormal expression of miRNAs is one of the important mechanisms of the occurrence and development of DN (Ishii et al., 2020), and it may become a potential biomarker for predicting DN due to its specificity and stability in body fluids.

Mir-192 is one of the earliest miRNAs that could regulate DN-induced pathological pathways. It was highly expressed in renal tissue and played a key role in renal development and differentiation (Abdelsalam et al., 2019). Later experiments showed that mir-192 could promote the fibrosis of glomeruli and tubules (Mu et al., 2013). Putta et al. demonstrated that TGF-β1 increased mir-192 levels in cultured mesangial cells and the glomeruli of diabetic mice, while decreasing renal mir-192 resulted in decreased renal fibrosis and improved proteinuria (Putta et al., 2012). It was also observed that in the early stage of renal injury, mouse mesangial cells treated with TGF-β1 showed upregulation of mir-192 and collagen α-2(I), and the mechanism may be related to mir-192 downregulating the expression of Smad-interacting protein 1 (SIP1) and δEF1 (also called ZEB1) to control TGF-β1-induced type 1 collagen 2α (COL1A2) and participate in the development of matrix accumulation (Kato et al., 2007).

On the contrary, some opposite evidence indicated that low mir-192 expression could be detected in tubular cells cultured with high TGF-β1 and glucose, which is similar to the renal biopsy samples obtained from patients with DN under decreased eGFR and tubulointerstitial fibrosis (Kato et al., 2007; Wang et al., 2010). It was also confirmed in cohort clinical trials that mir-192 levels in patients with large amounts of albuminuria were significantly lower than those in patients with normal albuminuria (Ma et al., 2016). Interestingly, a study observed the expression level of mir-192 in EVs of patients with different albuminuria levels (*n* = 80). The results showed that mir-192 was positively correlated with albuminuria. The level of mir-192 in EVs of the microalbuminuria group was significantly higher than that of the normal albuminuria group and control group, and mir-192 could distinguish the patients with normal albuminuria from those with microalbuminuria (AUC = 0.802, *p* < 0.001). It is proved that the significant increase of EVmiRNAs expression has potential value in diagnosing early DN. The different results may be caused by the multiple effects of miR-192 in the kidney, and its anti-fibrotic and pro-fibrotic effects are obviously dependent on the different regulatory roles of cells and in different stages of the kidney disease (Jia et al., 2016). However, more evidence *in vivo* and *in vitro* is needed to determine the significance of mir-192 for DN early prediction.

It has been proved that the translation of superoxide dismutase and p21-activated kinase could be inhibited by mir-377 in blood, which could induce fibronectin accumulation in DN (Wang et al., 2008). A study that examined the levels of mir-377 in the blood showed that the expression of mir-377 increased in T2DM patients and the level of mir-377 was significantly related to the severity of albuminuria. Furthermore, the AUC of mir-377 was 0.851 for diabetic patients and healthy subjects, while the AUC of normal albuminuria and abnormal albuminuria was 0.711 (Al-Kafaji and Al-Muhtaresh, 2018). Therefore, blood mir-377 might also be a promising biomarker for DN.

In addition, the decrease of mir-16 and mir-451-5p in the kidney may downregulate the expression of IL-6 and MMP-9 and therefore contribute to the pathogenesis of DN (Li et al., 2014; Xu et al., 2014). Another experimental study showed that the level of mir-451-5p in the urinary exosomal (UE) increased gradually in diabetes induced by streptozotocin, while the rise of the miR-16 level was delayed comparatively (Xu et al., 2014). Therefore, the UE mir-451-5p might be a more sensitive biomarker of DN. Moreover, miR-200 families, including mir-200a, mir-200b and mir200c, and mir-429 and miR-141, are considered to play an important role in maintaining epithelial differentiation and have an anti-fibrosis effect (Bracken et al., 2015). However, more evidence is still needed to verify their value as early predictive biomarkers of DN.

### Mineral and Bone Metabolic Disorders Based on CKD

It has been acknowledged that most CKD patients have a high risk of bone and mineral metabolic disorders and other extraosseous complications. Kidney Disease Improving Global Prognosis (KDIGO) working group defined that CKD-induced generalized disorders of mineral and bone metabolism as chronic kidney disease-mineral and bone metabolic disorders (CKD-MBD), and the term renal osteodystrophy used specifically to describe CKD-MBD (Moe et al., 2006).

The most classic mechanism of CKD-MBD is secondary hyperparathyroidism caused by decreased renal function leading to phosphate retention, hyperphosphatemia, and hypocalcemia triad (Gutiérrez, 2010). Researchers also observed that with the change of eGFR, the fibroblast growth factor 23 (FGF 23) levels increased significantly before the changes in phosphate and parathyroid hormone (PTH) levels (Isakova et al., 2011). FGF 23 could bind to FGF receptor (FGFR) in the kidney and parathyroid gland through Klotho, followed by playing an essential role in vitamin D and phosphate metabolism (Kuro-o, 2010). In the early stages of CKD, high levels of FGF 23 attenuate hyperphosphatemia by increasing phosphate excretion in proximal renal tubules and inhibit the production of 1,25(OH)2D leading to a decrease in intestinal calcium absorption, which aggravates secondary hyperparathyroidism (Miyamoto et al., 2005; Isakova, 2012). Hyperparathyroidism caused by CKD forms the accumulation of PTH in the body, triggering the mobilization of calcium in bone and fibrous osteitis. Although bone histomorphometry is considered the gold standard for diagnosing CKD-MBD, its invasiveness, cost, limited availability of sample techniques, and evaluation result in limitations in the clinical application (Ott, 2008). Therefore, discovering novel biomarkers related to bone histomorphometry in blood or urine is essential for evaluating CKD progression and could provide effective guidance for clinical intervention.

#### Parathyroid Hormone

PTH, a single-chain hormone, is mainly produced in the parathyroid gland and is considered to be the most classical biomarker for estimating bone turnover by KDIGO. As a direct regulator of bone formation, it could play an essential role in vitamin D and phosphate metabolism (Ardawi et al., 2012). PTH could be metabolized into PTH fragments, which could be measured by different PTH assays (Smit et al., 2019).

A lot of research has confirmed the effectiveness of PTH as a biomarker of CKD-MBD. Monier et al. found that PTH-(1–84)/C-PTH fragment ratio could be a predictor for bone turnover (Monier-Faugere et al., 2001). A recent multicenter cross-sectional retrospective diagnostic trial involving 492 dialysis patients demonstrated the value of serum biomarker intact PTH (iPTH) could distinguish between high bone formation rate/bone surface (BFR/BS) and non-high BFR/BS (Sprague et al., 2015). In addition to iPTH, the different biological effects of PTH fragments provide a new target for accurate clinical diagnosis and treatment of CKD-MBD patients, but its diagnostic value in CKD-MBD needs more in-depth research (Chen et al., 2018). However, the oxidation in the CKD process, the low reactivity of parathyroid hormone, and the accumulation of inhibitory C-terminal fragments at the target tissue level affect the accuracy of PTH in evaluating CKD-MBD to a certain extent (Katherine et al., 2010; Evenepoel et al., 2016). Nevertheless, accurate determination of PTH is an important part of clinical management for patients with CKD-MBD.

#### Activin A

Activin A is a multifunctional cytokine, which is the most abundant transforming TGF-β family protein found in the bone matrix. SakaiR et al. found that the expression of activin A was related to bone resorption firstly (Sakai et al., 2000).

Previous studies have shown that activin A could stimulate skeletal growth and inhibit activin signals (Peng et al., 2018). Furthermore, it has been shown to enhance the activity of osteoclasts, and the inhibitory effect of ligand trap RAP011 on activin type IIA could inhibit osteoclast formation and bone remodeling in CKD diabetic mice *in vitro* (Sugatani et al., 2017). Another study demonstrated that using natural antagonists of activin A inhibin in mice could lead to an increase in bone mineral density (BMD) (Perrien et al., 2007). A recent cross-sectional study involving 104 patients with CKD found that activin A could reflect histomorphological parameters (BFR/BS, Acf, ObS/BS, and OcS/BS) of bone turnover similar to those of PTH and FGF-23. Interestingly, blood activin A levels in patients with CKD increased earlier than those in PTH and FGF-23, suggesting the role of activin A in evaluating early CKD-MBD development (Lima et al., 2019).

#### Tartrate-Resistant Acid Phosphate 5b

TRAP5b is an enzyme mainly derived from osteoclasts (Guañabens et al., 2019). It has been found that osteoclast-like cells produce TRAP5b in cell lines differentiated by exposure to an osteoblast-derived ligand receptor activator for NF-κB ligand (RANKL) (Lv et al., 2015). TRAP5b could affect the function of phosphate by separating it from the protein. Furthermore, it is the only available useful biomarker reflecting bone resorption in CKD patients regardless of CKD progression, hemodialysis, or peritoneal dialysis (Shidara et al., 2008; Vervloet and Brandenburg, 2017). However, the bone histomorphometric evidence for its reliability as a biomarker of CKD-MBD is still insufficient.

In addition to the biomarkers mentioned above, N-terminal propeptide of procollagen-1, bone-specific alkaline phosphatase, and C-terminal crosslaps of collagen-1 might also be of certain value in CKD-MBD (Vervloet and Brandenburg, 2017).

## Prognostic Biomarkers of CKD

As a multifactorial disease, risk factors of CKD could play different roles in different individuals and stages of the disease. This indicated that the discovery of prognostic biomarkers of CKD is crucial. Prognostic biomarkers could be used to predict the likelihood of the clinical outcomes regardless of the treatment. The development of prognostic biomarkers of CKD is an important task for nephrologists, and it helps improve the ability to identify patients with poor prognoses and improve risk stratification and CKD diagnosis.

### FGF23

FGF23 is a bone-derived phosphaturic hormone with the physiological function of reducing the reabsorption of phosphorus in the kidneys, reducing the secretion of renal active vitamin D, and participating in the metabolism of minerals. In the early stage of CKD, the elevated serum FGF 23 is an independent risk factor related to CKD progression, ERSD, cardiovascular complications, and even death in CKD patients in different stages. A low level of FGF 23, about 30 ng/L, could be detected in the blood circulation under normal physiological conditions. But in the ESRD stage, the level of FGF23 increased more than 100 times higher than normal. FGF23 cleavage process is affected in CKD progression. Excessive accumulation of FGF 23 C-terminal could be observed, and the ratio of FGF23 C-terminal to intact FGF 23 is increased. Excessively elevated FGF 23 disturbed calcium-phosphate metabolism significantly, which increases the risk of adverse events. The increase of FGF23 is one of the earliest signals suggesting that FGF23 has the characteristics of CKD progression biomarkers.

CVD is the main cause of death in CKD patients. Gutierrez et al. (2009) have shown that FGF23 is significantly related to the upgrade in the left ventricular mass index and left ventricular hypertrophy, which is significantly associated with cardiovascular risk. In addition, studies have found that elevated FGF 23 C fragments are independently associated with the risk of myocardial infarction (Seiler et al., 2010). For patients with coronary artery disease, elevated serum FGF 23 is related to higher mortality and cardiovascular events; even after traditional cardiovascular risk factors, the concentration of serum C-reactive protein and impaired renal function return to normal and this association still exists.

In recent years, many studies have been done on the mechanism of how FGF 23 is associated with the poor prognosis of CKD. Experiments on rat cardiomyocytes (Faul et al., 2011) proved that FGF23 could bind to FGFR independently of Klotho and act on the calcineurin/activated T cell nuclear factor signaling pathway, which leads to pathological hypertrophy of cardiomyocytes. FGF23 could enhance and activate the renin-angiotensin-aldosterone system (RAAS) by inhibiting the expression of angiotensin-converting enzyme in the kidney and this effect is independent of other bone mineral metabolism. Some studies (Gutiérrez et al., 2009; Faul et al., 2011) investigated that the activation of RAAS caused by FGF23 may be one of the mechanisms of left ventricular hypertrophy. The stimulation of this pathway by FGF23 could uniquely act on the FGFR 4 receptor in cardiomyocytes, then prevent part of the function of FGFR, inhibit myocardial hypertrophy-related genes, and slow down the process of myocardial hypertrophy and myocardial fibrosis (Grabner et al., 2015). In addition, some studies (Grabner and Faul, 2016) confirmed that the increased FGF23 and decreased Klotho could jointly affect cardiac remodeling and promote the progression of uremic cardiomyopathy. On the other hand, FGF23 could lead to myocardial fibrosis through β-catenin and TGF β (Hao et al., 2016). Large cohort studies showed that high levels of serum FGF 23 are an independent risk factor for new-onset atrial fibrillation (Mathew et al., 2014; Mehta et al., 2016).

Furthermore, elevated FGF23 could reduce the level of circulating active vitamin D, which is one of the mechanisms of low vitamin D in patients with CKD. Low vitamin D levels have also been shown to cause adverse events through various mechanisms, including hypertension, vascular calcification, increased propensity for infection, and the activation of the RAAS system. It was found that the injection of FGF 23 would cause a rapid decrease in erythropoietin, suggesting that FGF23 is also associated with renal anemia, one of the CKD complications (Mathew et al., 2014). As FGF23 is strongly associated with adverse outcomes in CKD, it would be a promising biomarker for risk prediction or, even more importantly, targeting FGF23 may be a strategy to improve CKD outcomes.

### Klotho

Klotho is a novel antiaging gene, which was identified by Kuro-o in 1997. It could encode the secreted form (sKlotho) and membrane-bound form (mKlotho) proteins (Liu et al., 2019). Among them, sKlotho is believed to be a main active form that could be detected in blood circulation. It is mainly involved in regulating calcium and phosphorus metabolism and exerts anti-fibrosis, anti-inflammatory, anti-oxidant, and other renal protective effects (Hu et al., 2016). Cross-sectional studies have shown that serum Klotho levels in CKD patients are related to the degree of renal damage (Liu et al., 2019); the worse the renal function, the lower the level.

The reduction of serum Klotho not only is related to kidney damage but also increases the risk of progression to ESRD and the risk of death in CKD patients. Studies have confirmed that Klotho protein could inhibit the apoptosis of renal tubular epithelial cells (Liu et al., 2015) and transdifferentiation (Liu et al., 2017) and act on the kidneys in an autocrine or paracrine manner to exert anti-inflammatory and anti-oxidant effects. Klotho could inhibit renal interstitial fibrosis caused by epithelial-to-mesenchymal transition and the apoptosis of distal tubule epithelial cells mediated by tissue endoplasmic reticulum stress. Moreover, the reduction of Klotho could cause factors such as oxidative stress, inflammatory response, and apoptosis to damage kidney function and promote the progress of CKD, then inducing the poor prognosis of CKD. In other words, upregulated Klotho could activate FOXO-mediated manganese superoxide dismutase that leads to facilitating the removal of reactive oxygen (Sun et al., 2019) and inhibiting the inflammation through suppressing the processes of nuclear factor-κB-mediated inflammatory (Zhao et al., 2011; Buendía et al., 2015). Furthermore, the level of renal angiotensinogen and angiotensin II could be reduced after Klotho supplementation, which contributes to the amelioration of fibrosis in diabetic and adriamycin nephropathy (Takenaka et al., 2017; Takenaka et al., 2019) by targeting TGFβ-1/Smads and WNT/β-catenin signaling pathways (Doi et al., 2011; Zhou et al., 2013; Takenaka et al., 2019). In addition, Klotho supplementation could inhibit renal fibrosis by suppressing the endoplasmic reticulum stress and epithelial-mesenchymal transition (Liu et al., 2015; Liu et al., 2017). With the advantage of the pleiotropic beneficial activities, Klotho could be a novel biomarker and treatment target for renal fibrosis (Zou et al., 2018).

### Uromodulin

UMOD is a glycoprotein, also known as the Tamm–Horsfall protein, and could be synthesized in epithelial cells. Recently, a genome-wide association study had indicated the association in single-nucleotide polymorphisms in the UMOD gene, which codes eGFR and UMOD (Olden et al., 2014). The mutations in the UMOD gene could result in the loss of the correct folding ability of the synthesized uromodulin polypeptide chain, which deposit in the endoplasmic reticulum and fail to synthesize uromodulin with regulatory functions. At the same time, the abnormal deposition of uromodulin could accelerate the renal tubules’ apoptosis that leads to the loss of nephrons and causing renal failure.

With the advantage of exclusive production by tubular segment in the kidney, UMOD could be considered as a biomarker with sizeable levels that could be detected in normal urine (Youhanna et al., 2014; Hammond et al., 2016). Observational studies in the general population had shown that the level of urinary UMOD is positively associated with the eGFR, markers of tubular transport, and kidney volume (Pruijm et al., 2016). A fraction of UMOD, produced in the thick ascending limb, could be released into the circulation (Scherberich et al., 2018) and the concentration of serum UMOD is approximately 1,000 times lower than urinary levels. It is positively associated with UMOD excretion and creatinine clearance in patients with CKD (Steubl et al., 2016). The level of UMOD could be considered a biomarker for tubule function, with the higher levels reflecting the higher tubule function.

High UMOD excretion leads to lower serum levels, which could activate the body’s immune function or enter the tissue interstitium, especially the damaged renal tubules, and can be combined with neutrophils to promote the synthesis of IL-8, which could induce mononuclear cells to secrete IL-1β and TNF-α, then increase the expression of IL-2 receptors and HLA class II molecules on the surface of lymphocytes, finally lead to inflammation, cause the deterioration of chronic kidney disease, and affect the prognosis of CKD patients (Kottgen et al., 2010; Satanovskij et al., 2017).

Recently, studies have indicated that the low level of serum UMOD could be an indicator for the prevalence of kidney disease and a predictor for the future decline of renal function. The risk for CKD progression is inversely associated with the levels of serum UMOD. Furthermore, it has been demonstrated that serum UMOD could predict mortality independently from eGFR at baseline (Delgado et al., 2017). The prediction of renal decline facilitated by evaluating serum UMOD is valuable that the decrease of kidney function is a marker for cardiovascular risk and mortality.

## Conclusion and Outlook

Due to the complex etiology of CKD and the varying speed of development during the hidden period of the disease, the lack of tools for early identification of CKD progress and the lack of effective intervention measures have resulted in CKD becoming one of the fastest-growing causes of death in the world. This article summarized the current CKD-related biomarkers (Table 1), which have great potential in the prediction, progress monitoring, and prognosis improvement of CKD and its complications. However, proteinuria and serum creatinine obviously could not be replaced at present, which indicates that the novel biomarkers still have some limitations. First, due to the complex factors affecting the progression of CKD, a single biomarker lacks sufficient diagnostic sensitivity and specificity to reflect all the complexity of the underlying pathophysiology and has limited clinical application value. Second, the individual differences of biomarkers are obvious, which are susceptible to the influence of many factors such as diet, environment, and sampling time, leading to misjudgment of the disease. Third, the high cost of biomarker detection and the inconsistent detection methods also limit the clinical application of the biomarkers. Therefore, CKD research guided by the discovery of new biomarkers has attracted more and more attention from scientists all over the world. With the continuous discovery of new signal networks and pathological mechanisms of CKD, the development of a set of biomarkers with high sensitivity and specificity for different stages of CKD and the joint evaluation of multiple biomarkers have become a research hotspot.

**TABLE 1:** Summary of the progression and prognosis biomarkers of CKD.

Biomarkers	Categories	Expression	Biological effects	Application	References
MMP-9	Endopeptidases	Increase	The inhibition of MMP-9 could reduce the infiltration of neutrophils and other inflammatory cells	Biomarker of renal fibrosis	Wang H et al. (2019b)
MMP-2	Endopeptidases	Increase	The increase of MMP-2 per unit of urine led to a decrease in eGFR (0.1 ml/min/m^2^) during the 38-month follow-up	Biomarker of renal fibrosis	Nadkarni et al. (2016)
MMP-7	Endopeptidases	Increase	Activate the Wnt/β-catenin signaling pathway after renal injury	Biomarker of renal fibrosis	Li et al. (2021)
MCP-1	Chemokine	Increase	The upregulation of MCP-1 was accompanied by the activation of IκB/NF-κB signaling in CKD patients with macroalbuminuria	Biomarker of renal fibrosis	Feng et al. (2019)
DKK-3	Glycoprotein	Increase	Regulated the signal transduction of the Wnt/β-catenin signal pathway and induce renal tubulointerstitial fibrosis	Biomarker of renal fibrosis	Lipphardt et al. (2019)
5-MTP	Tryptophan metabolite	Decrease	Attenuated the expression of the pro-inflammatory factor NF-κB p65 and its target gene products MCP-1 and COX-2	Biomarker of renal fibrosis	Chen D. Q et al. (2019a)
1-AP	Polycyclic aromatic hydrocarbon metabolites	Increase	Activated of the aryl hydrocarbon receptor signaling pathway	Biomarker of renal fibrosis	Miao et al. (2020)
1-HP	Polycyclic aromatic hydrocarbon metabolites	Increase	Activated of the aryl hydrocarbon receptor signaling pathway	Biomarker of renal fibrosis	Miao et al. (2021b)
IS	Indole derivatives	Increase	Activated RAS system, induced cell senescence and apoptosis, promoted EMT, thus accelerated the progression of fibrosis, renal dysfunction, and CKD	Biomarker of gut–kidney axis disorder	Kim et al. (2012); Sun et al. (2012); Han et al. (2018)
PCS	Transformation of cresol	Increase	Induced renal injury and fibrosis by inhibiting Klotho gene expression, activating RAS/TGF-β pathway, inducing EMT, and causing NADPH oxidase-driven ROS	Biomarker of gut–kidney axis disorder	Sun et al. (2012)
					Watanabe et al. (2013)
TMAO	Byproducts of bacterial metabolism	Increase	Induced oxidative stress by inhibiting the expression of the oxidative stress inhibitor SIRT1, increasing H2O2, and reducing SOD activity	Biomarker of gut–kidney axis disorder	Ke et al. (2018)
Gal-3	Beta-galactoside binding protein	Increase	Modulated kidneys pro-inflammatory effects, regulated growth, differentiation, and proliferation of the cells, and mediated aldosterone-induced fibrosis of the heart and blood vessels	Biomarker of CVD based on CKD	Calvier et al. (2013); Madrigal-Matute et al. (2014); Vergaro et al. (2016)
sST-2	IL-1 receptor	Increase	Highly associated with adverse outcomes in patients with CVD, acute and chronic heart failure, or even death	Biomarker of CVD based on CKD	Dieplinger et al. (2014); Savic-Radojevic et al. (2017)
GDF-15	TGF-β cytokine	Increase	GDF-15 might take part in tissue inflammation, oxidative stress, and injured cardiomyocyte repair and show anti-apoptosis and anti-hypertrophy effects	Biomarker of CVD based on CKD	Kempf et al. (2006)
CypA	Cytoplasmic protein	Increase	Acted as inflammatory mediators under the stimulation of oxidative stress, inflammation, and hypoxia and participated in the process of inflammation and apoptosis by affecting multiple processes of transcriptional signal transduction	Biomarker of DN based on CKD	Sherry et al. (1992); Kim et al. (2004); Suzuki et al. (2006)
Periostin	Stromal cell protein	Increase	Increased the expression of TGF-β that could directly promote EMT and stimulate ECM synthesis and therefore induce extracellular matrix deposition	Biomarker of DN based on CKD	Gordon et al. (2012)
MicroRNAs	Composed of 19–24 nucleotides	Increase/Decrease	Cleaved mRNA or suppressed the translation by interacting with the complementary sequence in the 3′-untranslated region of its mRNA target, followed by regulating gene expression	Biomarker of DN based on CKD	He and Hannon, (2004)
PTH	Single-chain hormone	Increase	Played an essential role in vitamin D and phosphate metabolism	Biomarker of CKD-MBD	Ardawi et al. (2012)
Activin A	Transforming TGF-β family protein	Increase	Stimulated skeletal growth and inhibited activin signal	Biomarker of CKD-MBD	Peng et al. (2018)
TRAP5b	Enzyme	Increase	Affected the function of phosphate by separating it from the protein	Biomarker of CKD-MBD	Lv et al. (2015)
FGF 23	Phosphaturic hormone	Increase	Acted on the calcineurin/activated T cell nuclear factor signaling pathway that led to pathological hypertrophy of cardiomyocytes	Prognostic biomarkers of CKD	Faul et al. (2011)
Klotho	Antiaging gene	Decrease	Inhibited the apoptosis of renal tubular epithelial cells and transdifferentiation and acted on the kidneys in an autocrine or paracrine manner to exert anti-inflammatory and antioxidant effect	Prognostic biomarkers of CKD	Liu et al. (2015); Liu et al. (2017)
UMOD	Glycoprotein	Increase	Combined with neutrophils to promote the synthesis of IL-8, induce mononuclear cells, secrete IL-1β and TNF-α, then affect the prognosis of CKD	Prognostic biomarkers of CKD	Kottgen et al. (2010); Satanovskij et al. (2017)

With the advancement of bioinformatics and multi-omics technology, in-depth research and development of ideal biomarker clusters at different stages of CKD could make it possible to construct a precise treatment system for CKD. At present, CKD and its complications metabolomics research reveals that the occurrence and development of CKD are related to dysfunctions such as amino acid metabolism, TCA cycle, and lipid metabolism (Chen et al., 2016; Wang Y. N et al., 2019; Eddy et al., 2020). Amino acids and lipid metabolites could be used as potential biomarkers for development. Proteomics research has found that 273 peptides were differentially expressed in the urine of CKD patients. The developed CKD273 classifier (Good et al., 2010; Verbeke et al., 2021) has great potential in the early diagnosis and prognosis of CKD. Large-scale clinical trials are currently underway to evaluate its diagnostic efficacy. In recent years, the development of single-cell transcriptomics technology is expected to improve our understanding of human biology more widely. The application of single-cell transcriptomes to clinical biopsy samples and cells in urine to the development of relevant cell biomarkers would improve the accuracy of diagnosis and help make personalized predictions for patients with kidney diseases (Stewart et al., 2020).

At present, how to screen out valuable biomarkers from a large amount of metabolic data quickly and accurately is still a difficult point. An effective biomarker for CKD would have the following attributes (Tummalapalli et al., 2016): 1) it could be detected in the early stage of the disease process; 2) it could prognosticate CKD progression and mortality; 3) it is cost-effective; 4) it is stable in sample solution; 5) it could be tested in clinical labs timely; 6) it could represent different pathophysiological pathways compared with traditional markers.

More attention should be paid to the development process of biomarkers. Through the integration of transcriptomics, proteomics, and metabolomics data, combined with histopathology-related data, through the construction of machine learning-based big data analysis methods, high-dimensional, descriptive, and quantitative CKD data could be generated for accurate screening prediction and prognostic biomarkers of different stages of CKD. On this basis, through the development of corresponding detection technology, the “real world” evaluation of biomarkers through long-term clinical follow-up and the clinical value of biomarkers are clarified through clinical data. The implementation of biomarkers in CKD is highly anticipated in the future because they could provide information about the mechanism of kidney disease and improve clinical practice. The ultimate goal would be to define accurate functions and molecular classification of CKD progression in the clinic and to target the disease process using the right drugs so as to correctly improve the diagnosis, prognosis and precise treatment of CKD patients.
